# Tailored Strategies for Mental Health Support: Distinctions-Based Approaches for the Red River Métis Community

**DOI:** 10.23889/ijpds.v9i5.1670

**Published:** 2024-09-18

**Authors:** Kyler Nault, Megan Deptuch, Colton Poitras, Lisa Rodway, Frances Chartrand, Olena Kloss

**Affiliations:** 1 Manitoba Métis Federation

## Objective and Approach

The study investigates attitudes of Red River Métis (RRM) Citizens towards existing mental health services to assess satisfaction levels and enhance support and accessibility. Employing a Community-Based Participatory Research and Collective Consensual Data Analytic Procedure (CBPR/CCDAP) framework, a survey was conducted during health consultations in 2022 and 2023. It explored participant perspectives on existing programming, service availability, and future needs. Analysis of participant responses, stratified by demographics, offers insights into mental health attitudes among the RRM Community.

## Results

A total of 144 RRM Citizens participated in the survey, reporting "addiction," "trauma," and "stress" as primary causes of mental health issues. Presently, only 20% (n = 29) utilize mental health services, with over 40% (n = 58) expressing dissatisfaction with available services. Moreover, more than 90% (n = 130) of Citizens emphasized “an urgent need for additional mental health services tailored to the RRM Community”. Common barriers to accessing mental health services included lack of awareness and financial constraints.

## Conclusion

The study highlights RRM Citizens’ dissatisfaction with existing services and inadequate awareness of available mental health services and their protocols, presenting significant barriers to accessing services. Further investigations are warranted to determine whether these challenges stem from communication discrepancies, service inadequacies, or a combination of both factors.

## Implications

Findings from the study inform the development of distinctions-based mental health services for the Red River Métis Community, enhancing access, engagement, and policy development to address mental health disparities.

